# Exercise and diet support in breast and prostate cancer survivors: findings from focus groups

**DOI:** 10.1007/s00520-024-08652-7

**Published:** 2024-06-18

**Authors:** Jack Dalla Via, Christopher R. Andrew, Brenton J. Baguley, Nina Stewart, Jonathan M. Hodgson, Joshua R. Lewis, Mandy Stanley, Mary A. Kennedy

**Affiliations:** 1 Nutrition and Health Innovation Research Institute, School of Medical and Health Sciences, Edith Cowan University, Perth, WA Australia; 2 Institute for Physical Activity and Nutrition, School of Exercise and Nutrition Sciences, Deakin University, Geelong, VIC Australia; 3 Radiation Oncology, GenesisCare, Perth, WA Australia; 4 Medical School, The University of Western Australia, Perth, WA Australia; 5 School of Medical and Health Sciences, Edith Cowan University, Perth, WA Australia

**Keywords:** Exercise, Diet, Cancer, Supportive care, Survivorship

## Abstract

**Purpose:**

Cancer survival is improving, making optimal management of long-term treatment-related adverse effects increasingly important. Exercise and a healthy diet are beneficial and regularly recommended in cancer survivorship guidelines; however, few cancer survivors meet these recommendations so there is a need to explore why. This study aimed to understand experiences receiving exercise and diet support among Australian breast and prostate cancer survivors during and following treatment, and to explore what support they would like to receive.

**Methods:**

Adults who completed active treatment for breast or prostate cancer were recruited via a private cancer care centre. Using a qualitative descriptive study design, participants attended in-person focus groups that were recorded, transcribed, then analysed using reflexive thematic analysis.

**Results:**

In total, 26 cancer survivors (15 breast, 11 prostate) participated in one of seven focus groups (4 breast, 3 prostate). Two themes were developed: 1) *It was just brushed over*, and 2) *Wanting more*. Theme 1 reports that exercise, and especially diet, were rarely discussed. If they were, it was often limited to general recommendations. Theme 2 shows that participants wanted more specific and personalised support, and information about how exercise and/or diet could benefit their cancer treatment.

**Conclusion:**

Despite strong interest in receiving personalised exercise and diet support, neither are routinely provided to Western Australian breast and prostate cancer survivors. If support was provided, there was inconsistency in the level and type of support provided. These findings identify important gaps in exercise and diet support provision to cancer survivors and will inform future strategies aiming to improve cancer survivorship care.

**Supplementary Information:**

The online version contains supplementary material available at 10.1007/s00520-024-08652-7.

## Introduction

Cancer incidence is increasing in Australia due to a growing and ageing population, with breast and prostate cancer being the first and third most commonly diagnosed, respectively [1, 2]. Concurrently, cancer survival rates are also improving meaning more cancer survivors are living longer following a diagnosis [1]. This is especially true for breast and prostate cancers in which 92% and 96% of women and men, respectively, are expected to live at least 5 years following a diagnosis in Australia [1]. As a result, it is becoming increasingly important to consider how to best manage both short and long-term adverse effects of cancer treatment. For certain common cancer therapies, including chemotherapy, hormone therapy, and radiotherapy for certain cancer types, long-term treatment-related adverse effects can include accelerated musculoskeletal decline and/or increased cardiovascular disease risk [3, 4].

Regular exercise and a healthy diet are commonly recommended for cancer survivors, reflecting the wealth of evidence demonstrating wide-ranging benefits for many physical and psychosocial treatment-related adverse effects [5, 6]. Strong evidence indicates that exercise with or without dietary interventions can ameliorate long term adverse effects into cancer survivorship [5, 6], as well as reducing cancer-specific and all-cause mortality [7]. Healthy dietary patterns that consistently represent high intakes of fruits and vegetables with low processed foods and meats, have shown reduced cancer-specific mortality [6], particularly for breast cancer [8–10]. Individualised dietary interventions in breast or prostate cancer show significant benefits in weight loss and improving quality of life post-treatment or during hormone therapy [11–13]. Despite the evidence, the delivery of evidence-based exercise and nutrition information to cancer survivors is suboptimal [14, 15]. Cancer survivors in England participating in a focus group study reported receiving little to no advice on exercise or physical activity from healthcare professionals [15]. Similarly, a recent scoping review showed that Australian cancer survivors commonly reported limited or ineffective dietary information from healthcare providers following treatment, and that the role of diet in recovery from treatment was not often explained [14]. This is consequential as cancer survivors are more likely to make positive exercise and dietary changes if their oncology providers discuss these areas during their visits [16]. Furthermore, several health care related barriers to engaging in both exercise and nutrition services that have been reported, including the cost, availability, and access to cancer-specific services, lead many survivors to make lifestyle changes without allied health support [17, 18]. To address the many evident barriers and successfully change the lifestyle of a cancer survivor, various behaviour change frameworks emphasise the need to consider multiple factors, such as capability, opportunity and motivation in the COM-B model of behaviour change [19]. Importantly, exercise and dietary support provided to cancer survivors has typically been explored separately, so whether issues affecting provision of this support are common or unique remains unclear.

To achieve the goal of routine delivery of exercise and diet support for people with cancer, it is important to understand how to improve current practices. Therefore, the aim of this study was to understand breast and prostate cancer survivors experience and perspectives on exercise and diet support provided to them during and following treatment, and to explore what support cancer survivors would like to receive. The findings will inform future work to integrate effective exercise and diet support into cancer care.

## Methods

### Study design

A qualitative descriptive study design was chosen as an appropriate approach to gain the perspective of people with relevant lived experience [20]. Taking a relativist ontology and a subjectivist epistemology the researchers value the perspective of the participants and ensure that in the interpretation their perspective is foregrounded [21]. Data collection was through focus groups, chosen to allow for open discussion and group interaction, which may help to uncover insights that would not be accessible otherwise [22]. Ethical approval was granted by the Edith Cowan University Human Research Ethics Committee (Project number 2021–02863-DALLAVIA). The study is reported in accordance with the consolidated criteria for reporting qualitative research (COREQ) framework [23] for reporting qualitative research.

### Study sample

#### Eligibility

Eligible participants were aged 18 or older and had completed active treatment (ongoing hormone therapy acceptable) for breast or prostate cancer with therapies known to have cardiovascular adverse effects. For breast cancer, this included radiotherapy, chemotherapy and/or aromatase inhibitor therapy [24]. For prostate cancer, this included androgen deprivation therapy and/or chemotherapy [25]. These criteria were due to a secondary aim of the study (to be reported separately) to explore cancer survivors’ understanding of how cancer treatment may affect their cardiovascular and musculoskeletal health.

#### Recruitment

Participants were recruited for the study using purposive sampling, facilitated by a large private cancer care provider in Perth, Western Australia. An invitation email explaining the study was sent to 354 potential participants (183 breast cancer survivors, 171 prostate cancer survivors) who had completed active treatment from July 2020 to June 2021. Email recipients were asked to contact the lead investigator if interested in participating. All interested participants were screened for eligibility via telephone in October and November 2021, and eligible participants provided informed consent prior to completing the preliminary online questionnaire.

### Data collection

#### Questionnaire

Background information about participants was collected using an online questionnaire (Qualtrics, Utah, USA) completed prior to attending the focus group. The questionnaire asked participants to self-report demographic (i.e., gender, marital status, ethnicity, education and occupation) and medical information (general medical history as well as cancer diagnosis and treatment information). Additionally, open-ended and closed questions about physical activity and diet behaviours prior to, during, and following cancer treatment were included. Participants were provided with a brief overview of Australian physical activity and dietary guidelines for healthy adults, then asked whether or not they were meeting these recommendations prior to their cancer diagnosis, and why they thought they were/were not. Participants were then asked whether their physical activity levels or diet improved, stayed the same, or declined during and following cancer treatment, and why this was so.

#### Focus groups

Focus groups were conducted in person at the Royal Perth Hospital Research Foundation building in Perth in December 2021. Breast and prostate cancer survivors participated in independent focus groups in order to determine specific experiences within each cancer type and to help participants feel comfortable sharing their experiences. All participants who attended a focus group received a $30 (AUD) gift voucher as reimbursement for their time and any travel expenses incurred. All focus groups were moderated by the lead investigator (JDV), who is a male postdoctoral research fellow with experience conducting research with cancer survivors. An additional observer was present in each focus group to record field notes to provide contextual information not captured by the audio recording [26]. Only the moderator (JDV), observer and participants were present in the room for each focus group. A focus group guide was developed collaboratively by the research team (question list provided as supplementary information). This was used to guide the conversation during each focus group, with the moderator asking follow-up questions or additional questions where necessary to explore, clarify or obtain further information based on participant responses. For practical reasons, the focus group guide was not piloted prior to the study, but small changes were made after the first focus group to ensure the questions were worded clearly and that the discussions covered important aspects of the topic. The moderator began each focus group with refreshments before moving to formally introduce the researchers present and the aim of the study. Each focus group was digitally recorded, then professionally transcribed (Digital Transcripts, Victoria, Australia).

### Data analysis

Closed, forced-choice demographic, medical, and physical activity and diet questionnaire responses were summarised descriptively. Focus group transcripts were checked for completeness and accuracy by the moderator (JDV) then imported into NVivo (version 12, QSR International) for analysis. They were not returned to participants for comment, for practical reasons and considering they were transcribed verbatim. Focus group data were analysed using reflexive thematic analysis following Braun and Clarke’s 6 steps [27, 28]. Four authors were involved in the coding of focus group transcripts (JDV, MK, CA, MS). Initially, two authors (JDV, CA) familiarised themselves with each transcript, then each coded the same focus group transcript to check for consistency in analysis. Their coding was compared and discussed with other authors (MK, MS) who have extensive qualitative research expertise, to check and decide on a consistent approach to use for the remaining transcripts. The two authors then analysed the remaining focus groups, collated the codes into categories, and extracted relevant data. These categories were shared with other authors (MK, MS) who provided feedback and encouraged further reflection on how the codes should be best categorised. Next, the two authors generated initial themes from the categories, which were reviewed and further developed by discussion among the authors. This ongoing process helped to refine the themes to ensure they appropriately represented the data, answered the research question, and had an informative name. The participants did not provide feedback on the findings. The moderator (JDV) has a background in exercise science and nutrition research, and this was disclosed to participants when the moderator introduced themself at the beginning of each focus group. The other authors have backgrounds in health, implementation science, oncology, exercise physiology and dietetics, and include a range of expertise in the use of qualitative and quantitative methodologies.

## Results

### Study sample

**Fig. 1:**
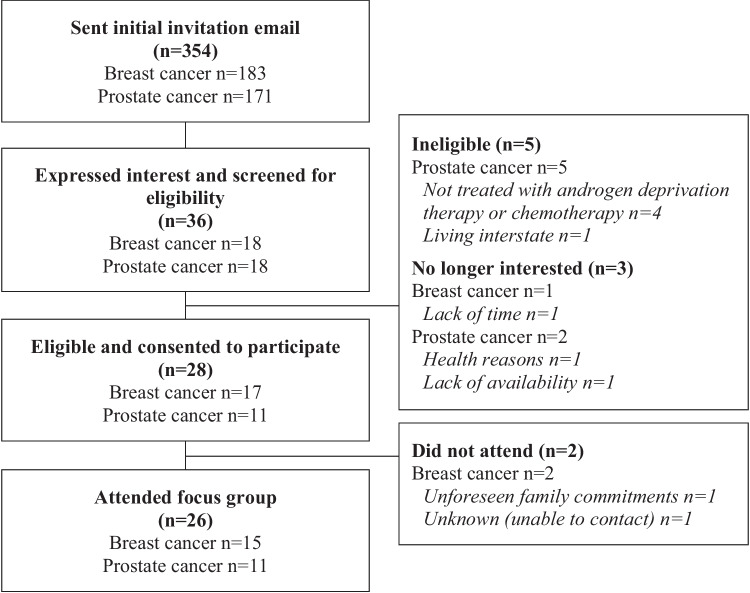
Study recruitment flowchart

A total of 26 cancer survivors (15 breast cancer, 11 prostate cancer), with a mean ± SD age of 66.7 ± 11.7 years, participated in a focus group. Details of the study recruitment process are presented in Fig. 1. Seven focus groups (four with breast cancer survivors, three with prostate cancer survivors) each consisting of three or four participants were completed, ranging from 54 to 107 min in duration. Characteristics of the final sample are presented in Table 1.

**Table 1:** Participant characteristics

Demographics	Breast cancer (*n* = 15)	Prostate cancer (*n* = 11)
Age	61.2 ± 11.6	74.2 ± 7.0
Gender	Female: *n* = 15 (100%)	Male: *n* = 11 (100%)
Ethnicity		
Caucasian	13 (87%)	11 (100%)
Asian	2 (13%)	0 (0%)
Marital status		
Married	9 (60%)	8 (73%)
De facto	3 (20%)	1 (9%)
Divorced/separated	3 (20%)	0 (0%)
Widowed	0 (0%)	1 (9%)
Single	0 (0%)	1 (9%)
Highest education level		
Completed secondary school	6 (40%)	2 (18%)
Technical/trade qualification	0 (0%)	2 (18%)
Diploma/certificate	5 (33%)	2 (18%)
Tertiary degree	4 (27%)	5 (46%)
Employment		
Employed full-time	2 (13%)	3 (27%)
Employed part-time	7 (47%)	2 (18%)
Retired	3 (20%)	6 (55%)
Full time home duties, carer	3 (20%)	0 (0%)
**Cancer diagnosis and treatment**		
Cancer stage		
Stage I	6 (40%)	0 (0%)
Stage II	3 (20%)	2 (18%)
Stage III	3 (20%)	0 (0%)
Unknown	3 (20%)	9 (82%)
Time since diagnosis (months)		
Treatment(s) received		
Surgery	15 (100%)	1 (9%)
Radiotherapy	15 (100%)	11 (100%)
Chemotherapy	4 (27%)	0 (0%)
Hormone therapy	13 (87%)	11 (100%)
**Other chronic conditions**		
Respiratory disease	4 (27%)	3 (27%)
Angina/stroke/heart condition	0 (0%)	3 (27%)
Diabetes	0 (0%)	1 (9%)
Family history of heart disease	7 (47%)	4 (36%)
Hypertension	3 (20%)	8 (73%)
Hypercholesterolemia	4 (27%)	7 (64%)
Cancer (other than breast or prostate)^a^	1 (7%)	0 (0%)
Osteoporosis	0 (0%)	1 (9%)
Arthritis	4 (27%)	5 (46%)
Fracture	3 (20%)	3 (27%)
Recent fall (last 12 months)	0 (0%)	1 (9%)

Data are mean ± SD or *n* (%). ^a^excluding skin cancer

### Physical activity and diet behaviours

Responses to the closed physical activity and dietary behaviour questions in the online questionnaire are presented in Table 2. Most prostate cancer survivors (82%), but not breast cancer survivors (40%), self-reported meeting physical activity guidelines prior to cancer treatment. Conversely, most breast (73%) but not prostate cancer survivors (46%) reported adhering to dietary recommendations prior to treatment. After diagnosis and during treatment, most cancer survivors decreased their physical activity levels (65%), while dietary changes were mixed. More breast than prostate cancer survivors increased their physical activity levels (53% vs 27%) and improved their diet (47% vs 27%) following treatment.

**Table 2:** Self-reported physical activity and diet behaviours

	Breast cancer (*n* = 15)	Prostate cancer (*n* = 11)
**Physical activity behaviours**		
Meeting physical activity guidelines prior to cancer treatment		
Yes, *n* (%)	6 (40%)	9 (82%)
No, *n* (%)	9 (60%)	2 (18%)
Change in physical activity following diagnosis/during treatment		
Increased, *n* (%)	1 (7%)	0 (0%)
Maintained, *n* (%)	4 (27%)	4 (36%)
Decreased, *n* (%)	10 (67%)	7 (64%)
Change in physical activity since completing treatment (compared to during treatment)		
Increased, *n* (%)	8 (53%)	3 (27%)
Maintained, *n* (%)	4 (27%)	4 (36%)
Decreased, *n* (%)	3 (20%)	4 (36%)
**Diet behaviours**		
Following recommended dietary pattern prior to cancer treatment		
Yes, *n* (%)	11 (73%)	5 (46%)
No, *n* (%)	4 (27%)	6 (55%)
Change in diet following diagnosis/during treatment		
Became healthier, *n* (%)	7 (47%)	3 (27%)
Stayed the same, *n* (%)	4 (27%)	5 (45%)
Became less healthy, *n* (%)	4 (27%)	3 (27%)
Change in diet since completing treatment (compared to during treatment)		
Became healthier, *n* (%)	7 (47%)	3 (27%)
Stayed the same, *n* (%)	5 (33%)	5 (45%)
Became less healthy, *n* (%)	3 (20%)	3 (27%)

### Themes

Two themes were developed and are described below. The findings are supported with direct quotes from participants, presented below in italics, deidentified using a participant number, focus group number, cancer type, and participant age. Experiences were largely similar for breast and prostate cancer survivors, so the themes and discussion points below apply to both participant groups unless otherwise specified.

#### Theme 1: It was just brushed over

There was some variation in the level of support provided to cancer survivors during and following treatment, however participants most consistently reported receiving minimal exercise and diet support. This was despite participants consistently reporting that they would have and guidance.

Nobody spoke to me about diet or exercise. Not at all. (Participant [P] 17, Focus group [FG] 5, Prostate cancer, Aged 67 years)

I think it [diet and exercise] was just brushed over (P9, FG1, Breast cancer, Aged 66 years)

I wanted some guidance to give me the opportunity to address [my diet] and improve my health. but it wasn’t covered at all in my case. (P10, FG2, Breast cancer, Aged 34 years)

This was in contrast to one participant who received a thorough survivorship plan that incorporated advice about diet and exercise, and links to websites for further reading or to access additional support.

he said ‘now I’m giving you a survivorship care plan’ (P15, FG2, Breast cancer, Aged 65 years)

For those who did receive exercise and/or diet support, it was primarily provided as general advice. Participants were often told to be physically active and to eat well, but not offered any specific support on what to do or how to achieve this.

They just said, ‘Do you do any exercise?’ I said ‘yeah’, and they said, ‘You know you’ve got to diet?’ and I said, ‘Yeah, well, I know that. That’s common sense anyway.’ But they didn’t sort of push it” (P16, FG6, Prostate cancer, Aged 71 years)

They just say, ‘You need to keep your diet, you know, you need to have good dietary practice. You need to get some exercise.’ That’s all they say. (P20, FG6, Prostate cancer, Aged 78 years)

But it was very basic, very, very basic… (P14, FG4, Breast cancer, Aged 60 years)

It was clear that participants were not told about why regular exercise and maintaining a healthy diet is important for their cancer specifically. Some participants were told to be physically active and eat well; however, the message did not differ from general health advice for people without cancer.

We certainly all know why exercise is important, and diet, but whether it’s more important because you have cancer, I don’t know that (P23, FG5, Prostate cancer, Aged 81 years)

exercise and diet are so important, cool, it has been all my life but there’s no why… If they said ‘You need to exercise because it stimulates whatever it is and it’s going to help your body recuperate’, cool, but they just say exercise, you know? (P22, FG7, Prostate cancer, Aged 63 years)

Participants often reported receiving written information about exercise and diet; however, this was provided without discussion and many participants did not read the information thoroughly or were overwhelmed by the volume of it.

They also gave me a book about this thick and a whole pile of pamphlets and things which I kind of flicked through, but I really didn't read them. (P6, FG3, Breast cancer, Aged 75 years)

Most participants felt that this written information needed to be supported by a discussion with someone in the medical team.

It's all on a pamphlet about what you should do, but I think if somebody sat down and talked it through with you properly, you might be more inclined to do it. (P8, FG1, Breast cancer, Aged 65 years)

The provision of support or advice related to exercise and diet was often reactive rather than proactive. Participants reported that they only received support if they initiated the discussion or asked their medical team for it. Some participants perceived this to mean their medical team did not think they needed additional support.

Maybe if I need it they would send me (for exercise or diet intervention). But I don't know; nobody sent me, so… (P13, FG3, Breast cancer, Aged 63 years)

Consequently, attempts to change exercise and/or dietary behaviours were typically self-initiated rather than being due to support or advice provided to them as part of their cancer care.

The minute I found out [about cancer diagnosis] I changed my diet (P2, FG3, Breast cancer, Aged 52 years)

I just want to do the best I can for my body. I'm not a surgeon. I'm not a doctor. I can't do any of that. But I can look after myself. So that was what inspired me to just live my healthiest life for me. (P2, FG3, Breast cancer, Aged 52 years)

#### Theme 2: Wanting more

There were many aspects of exercise and dietary support that left participants wanting more, and they provided ideas and suggestions about how support could be improved to be better suited to their needs. Understanding the “why” may play an important role in motivating cancer survivors to achieve healthful behaviour change. Most participants reported that understanding why exercise and diet can help them with their cancer would be a strong motivator for them to change their behaviours.

I suppose if they told you why, you might be motivated a bit more to do it. (P23, FG5, Prostate cancer, Aged 81 years)

Just the why; for me it would have been the why. Why should - how is it going to help us? (P2, FG3, Breast cancer, Aged 52 years)

This was evident for one prostate cancer survivor who reported exercising consistently during radiotherapy and was asked what motivated him to do so.

the fact that they told me that we’d get a better outcome if I did exercise. Um, what they did say is it was good to do the exercise directly before you had the radiation (P26, FG6, Prostate cancer, Aged 68 years)

Most participants wanted exercise and diet support that was individually tailored to their preferences and personal circumstances. Further to this, participants identified a broad range of considerations that they perceived to be important when personalising exercise and diet support. This included discussions about tailoring support based on their pre-existing knowledge or experience with exercise, their preferences and health status, their work and family circumstances, and motivation. The common considerations discussed, along with examples and representative quotes, are summarised in Table 3.

**Table 3:** Considerations for tailoring exercise and diet support raised by participants

Consideration	Example(s)	Representative quotes
Health conditions (cancer and non-cancer related)	Some participants had other health conditions or were experiencing particular treatment-related side effects, so wanted exercise prescription or dietary support that was individualised based on this	*“Like [he] falls over, I can’t sit on a bike because of my back and my knees. That’s where you really need to sit with somebody that can tailor that and say ‘Okay, you have these issues, let’s do a plan that makes sure [he] doesn’t go face down on the pavement’ or … ‘[I’m] not in excruciating pain’. That would really be helpful.”* (P22, FG7, Prostate cancer, Aged 63 years) * “I didn’t know what [exercise] I could do. What I couldn’t do.”* (P11, FG2, Breast cancer, Aged 46 years) * “I used to go to the gym all the time, but I was exhausted.”* (P9, FG1, Breast cancer, Aged 66 years) * “my appetite never came back properly.”* (P12, FG4, Breast cancer, Aged 61 years)
Knowledge and experience	Some participants wanted more specific exercise and dietary support that included clear information about what exercise they should be completing or what they should or should not eat	*“One of the things that would have been helpful, instead of saying exercise, what sort of exercise?… You tell me there’s a scientific link, it’s going to make me feel good, but what am I supposed to do?”* (P22, FG7, Prostate cancer, Aged 63 years) * “So diet would be great, just to know what should we eat, and what shouldn't we eat, does it matter? does it affect us?”* (P2, FG3, Breast cancer, Aged 52 years) * “I don’t know whether they expect you to know [what to eat or not eat]”* (P1, FG2, Breast cancer, Aged 76 years)
	Other participants who already exercised regularly prior to their diagnosis felt that the information provided to them would be more appropriate for people who were previously inactive, so wanted support that was more relevant to them	*“The things that you were sort of given were very much focused on people who were probably inactive firstly, whereas people like ourselves who are very active prior, there was really nothing like to guide us on – Okay, so what do we do now when you are previously a very active person?”* (P11, FG2, Breast cancer, Aged 46 years) * “they gave me a booklet and things to do with this rubber band, you know? You tie it to the wall and this and that. But I was doing all that at the gym, so I didn’t want to do that”* (P22, FG7, Prostate cancer, Aged 63 years)
Preferences	Consideration for individual preferences regarding diet and types of exercise were an important consideration for some participants	*“I wouldn't do the gym, I think that would be a good thing but I don't like the gym. They recommend dancing, so I would do that”* (P8, FG1, Breast cancer, Aged 65 years) * “I mentioned that I had an exercise bike and they said that’d be good to get on the exercise bike but that I find totally boring so I’ve since given it away”* (P18, FG7, Prostate cancer, Aged 79 years)
Work commitments	Participants who were working often reported that limited time outside of work, or the nature of their job meant they did not exercise regularly	*“I was leaving for work at 6:30 and getting home at 6:30. So, I didn’t do much exercise.”* (P12, FG4, Breast cancer, Aged 61 years) * “That’s my own personal habit, I don’t exercise. I don’t like to exercise. Maybe it’s my job. My work itself is really very strenuous.”* (P15, FG2, Breast cancer, Aged 65 years)
	Work also impacted participants ability to attend a hospital-based exercise program	*“I saw the flyer [about a hospital exercise program], but because I kind of work I can't go during the times that it's on.”* (P5, FG1, Breast cancer, Aged 50 years)
Family commitments	Family circumstances were a common challenge to a healthy diet, particularly among several breast cancer survivors who did the cooking for their family and had to consider all of their preferences	*“Yeah if you're cooking for a family it's difficult”* (P9, FG1, Breast cancer, Aged 66 years)
	Conversely, some men with prostate cancer suggested dietary support would not be useful for them as they do not cook for themselves	*“Well, [diet support] not going to do any good with me. I don’t cook.”* (P23, FG5, Prostate cancer, Aged 81 years)
Motivation	Participants often reported a lack of motivation to change their behaviour, despite knowing that it would be beneficial to do so	*“The thing is, I have read all that information and I know it all but as I said, that’s my failing. I can read all I want but that doesn’t mean I’m going to do it.”* (P21, FG5, Prostate cancer, Aged 69 years) * “I’m well aware of all the health benefits… and I know it’s what I should be doing… I need to get someone to get me motivated, that’s my problem at the moment, lack of motivation”* (P18, FG7, Prostate cancer, Aged 79 years) * “for me if someone, you know, encouraged me and gave me the option to join a group or something to exercise while I'm so lazy to exercise, that will make me”* (P13, FG3, Breast cancer, Aged 63 years)
Ongoing support	Even participants who did improve their exercise or diet behaviours were not able to sustain the changes long-term, so desired ongoing support	*“It was healthy food but it tasted like shit, so… we ate it for a while and it worked pretty good but there’s a limit for how long you can do it for.”* (P19, FG5, Prostate cancer, Aged 72 years) * “What I found was that I did exercises conscientiously while I was having the radiation therapy and then my work commitments and that feeling of lethargy kind of dragged me down”* (P26, FG6, Prostate cancer, Aged 68 years)

Finally, most participants reported that they would have taken up a referral to an allied health professional to help with exercise (e.g., an exercise physiologist) or with diet (e.g., a dietitian), however very few received a referral. One participant independently sought support from an exercise physiologist.

I would like to be referred to someone… to help me with everything, like, how to exercise, what kind of exercises. (P13, FG3, Breast cancer, Aged 63 years)

I’d take it. If they said, ‘Here’s a referral. We would like you to go and see a dietitian’, we’d go and see a dietitian. (P17, FG5, Prostate cancer, Aged 67 years)

I sourced one [an exercise physiologist] privately. (P13, FG2, Breast cancer, Aged 46 years)

## Discussion

This study presented findings from focus group discussions with breast and prostate cancer survivors. Their experience of and preferences for receiving exercise and dietary support during and following treatment were explored. Findings demonstrated that exercise, and especially diet, were rarely discussed in any depth, if at all. Moreover, participants wanted more specific and tailored support that considered personal preferences, circumstances, and capabilities, and most would welcome referrals to allied health professionals who are best placed to provide this support. Participants also agreed that more specific information about how exercise and/or diet would benefit their cancer treatment would motivate them to act.

Despite a strong preference for tailored information and support about exercise and diet, most participants reported a lack of specific information. All participants in our study were receptive to receiving information and support about exercise and diet during treatment. This finding aligns with available literature in cancer survivors demonstrating an interest in personalised support to help people feel informed about what to do, as well as providing practical strategies to improve behaviours [14, 15, 29–33]. In particular, a systematic rapid review of 118 studies in 15 cancer types exploring factors influencing physical activity concluded that physical activity support and advice should be individualised, considering patient-specific needs and preferences [29]. Similarly, in a scoping review of dietary information provision in Australia, cancer survivors reported receiving limited or ineffective dietary advice, and identified a need for individualised dietary strategies and practical skills for healthy eating [14]. Many of the specific considerations for tailoring exercise and diet support participants in our study discussed are also consistent with those identified by cancer survivors in previous qualitative studies [15, 31–33]. Importantly, several common issues and barriers were identified in the provision of both exercise and dietary support, and preferences for support delivery expressed by participants was largely consistent for exercise and diet. Taken together, our results and the available literature show a clear need to tailor both exercise and diet support to each individual cancer survivor.

There is an evident need to consider ways to support behaviour change more effectively in cancer survivors. The COM-B model identifies capability, opportunity and motivation as key factors contributing to behaviour change [19], and participants identified factors relating to each when discussing what would help them engage in exercise or healthy eating. The desire for individually tailored exercise and diet support also reflects qualitative evidence that cancer survivors value free choice and autonomy when changing behaviour [34]. Our finding that cancer survivors would be more motivated to exercise and eat well if they understood why it was beneficial for managing their cancer diagnosis is important as a lack of motivation was a common consideration identified by participants. This aligns closely with the aforementioned systematic rapid review, which identifies patient education about the importance of physical activity post-cancer, as well as the incorporation of behaviour change strategies to increase physical activity levels among cancer survivors [29]. Strategies to specifically address a lack of motivation among cancer survivors, such as motivational interviewing, have shown promising effects on various lifestyle behaviours including physical activity and diet [35]. However, systematic reviews also report that approximately 41–43% of combined exercise and nutrition interventions in cancer survivors did not incorporate any behaviour change techniques or theories [36, 37]. Overall, this study further highlights the need to explore practical strategies to effectively leverage individual motivation into successful behaviour change.

Implementation issues commonly underpin provision of exercise and diet support in cancer care and should be considered in working toward solutions. Data suggest the lack of support is not due to a lack of interest: two oncology clinician surveys reported approximately 80% of respondents recognised exercise as an important part of cancer care [38, 39]. Instead, their challenge is that they do not feel adequately prepared to deliver this information. Barriers identified by oncologists in Australia include a lack of exercise or nutrition-specific knowledge or training, as well as a lack of time within consults to discuss exercise and nutrition with their patients in addition to essential oncology-specific information [40, 41]. While this suggests referral to allied health for dedicated exercise and/or nutrition support consults may be an appropriate solution, the current referral process was also identified as a barrier [40, 41]. The lack of effective infrastructure within health care organisations was highlighted as the most commonly reported barrier in a recent scoping review of implementation barriers to delivery of exercise oncology services [18]. The lack of clear referral pathways and knowledgeable workforce to deliver tailored support likely underpins the evident lack of referrals to allied health professionals (e.g., exercise physiologists, dietitians) reported in the study. In order for these supportive care services to be useful, a clear strategy for how to integrate them into care in a way that is accepted by an organisation is critical. Therefore, there is a need for focused implementation work to overcome these referral barriers, so that cancer survivors can be connected with the allied health professionals best placed to provide the specific and tailored support they desire. The consistency in current issues and desired support for both exercise and diet mean that implementation work can be readily adapted across the various allied health services accessed by cancer survivors. Overcoming these common barriers may therefore improve multiple lifestyle aspects, including promoting sustained exercise and dietary behaviour change.

A number of limitations should be considered when interpreting the study results. The inclusion of breast and prostate cancer survivors means the findings may not be transferable to other cancer populations. Recruitment was facilitated by one service so all participants were patients at one of their centres in Western Australia, possibly introducing recruitment bias. The provider is a private cancer care provider, so findings may not be transferable to the wider population of cancer survivors treated exclusively in the public system. While all participants received radiation therapy from the same private cancer care provider, they may have received other treatments (e.g. surgery, chemotherapy, hormone therapy) at other hospitals or cancer care centres, so their reported experiences could potentially reflect various healthcare settings. Participants were recruited from various treatment centres across Western Australia but were required to attend a focus group in person in the Perth city, so are less likely to include rural cancer survivors. Additionally, while qualitative research is context specific and the results may not be directly generalisable to cancer survivors in other countries, the issues assessed through the focus group questions reflect the needs raised through research in countries that share similar demographic characteristics and/or health care systems. There is a risk of those opting into the study more likely to have an interest in exercise and diet. Background information about participants was obtained from a self-report questionnaire, which led to incomplete data on cancer stage for several participants and meant responses to certain questions may be subject to recall bias or under/overestimation. While the moderator made a conscious effort not to contribute personal thoughts and knowledge to the discussions, it is possible that this had some influence. A relationship between the moderator and participants was not established prior to the study commencing, other than during telephone conversations as part of the recruitment and screening process.

In conclusion, exercise and diet support are currently not routinely incorporated into the care of Western Australian breast and prostate cancer survivors, with wide variation in the level of support provided. The current findings suggest survivors are extremely receptive to receiving support. Strategies that can tailor exercise and diet support to the specific cancer benefits and circumstances of individual cancer survivors, using behaviour change techniques, would be widely accepted and have the potential to improve exercise and diet behaviours among cancer survivors.

### Supplementary Information

Below is the link to the electronic supplementary material.

Supplementary file1 (DOCX 19 KB)

## Data Availability

The data that support the findings of this study are available from the corresponding author upon reasonable request.

## References

[1] Australian Institute of Health Welfare (2021) Cancer in Australia 2021. Cancer series no. 133. Cat. no. CAN 144. AIHW, Canberra

[2] Sung H, Ferlay J, Siegel RL, Laversanne M, Soerjomataram I, Jemal A (2021). Bray F Global cancer statistics 2020: GLOBOCAN estimates of incidence and mortality worldwide for 36 cancers in 185 countries. CA Cancer J Clin.

[3] Okwuosa TM, Anzevino S (2017). Rao R Cardiovascular disease in cancer survivors. Postgrad Med J.

[4] Sturgeon KM, Mathis KM, Rogers CJ, Schmitz KH (2019). Waning DL cancer-and chemotherapy-induced musculoskeletal degradation. JBMR plus.

[5] Campbell KL, Winters-Stone KM, Wiskemann J, May AM, Schwartz AL, Courneya KS, Zucker DS, Matthews CE, Ligibel JA, Gerber LH, Morris GS, Patel AV, Hue TF, Perna FM (2019). Schmitz KH Exercise guidelines for cancer survivors: consensus statement from international multidisciplinary roundtable. Med Sci Sports Exerc.

[6] Rock CL, Thomson CA, Sullivan KR, Howe CL, Kushi LH, Caan BJ, Neuhouser ML, Bandera EV, Wang Y (2022). Robien K American Cancer Society nutrition and physical activity guideline for cancer survivors. CA Cancer J Clin.

[7] Patel AV, Friedenreich CM, Moore SC, Hayes SC, Silver JK, Campbell KL, Winters-Stone K, Gerber LH, George SM (2019). Fulton JE American College of Sports Medicine roundtable report on physical activity, sedentary behavior, and cancer prevention and control. Med Sci Sports Exerc.

[8] Castro-Espin C (2022). Agudo A The role of diet in prognosis among cancer survivors: a systematic review and meta-analysis of dietary patterns and diet interventions. Nutrients.

[9] Jochems SHJ, Van Osch FHM, Bryan RT, Wesselius A, van Schooten FJ, Cheng KK (2018). Zeegers MP Impact of dietary patterns and the main food groups on mortality and recurrence in cancer survivors: a systematic review of current epidemiological literature. BMJ Open.

[10] Spei M-E, Bellos I, Samoli E (2023). Benetou V Post-diagnosis dietary patterns among cancer survivors in relation to all-cause mortality and cancer-specific mortality: a systematic review and meta-analysis of cohort studies. Nutrients.

[11] Mohamad H, McNeill G, Haseen F, N'Dow J, Craig LC (2015). Heys SD The effect of dietary and exercise interventions on body weight in prostate cancer patients: a systematic review. Nutr Cancer.

[12] Reeves MM, Terranova CO, Eakin EG (2014). Demark-Wahnefried W Weight loss intervention trials in women with breast cancer: a systematic review. Obes Rev.

[13] Umlauff L, Weber M, Freitag N, Fairman CM, Heidenreich A, Bloch W (2022). Schumann M Dietary interventions to improve body composition in men treated with androgen deprivation therapy for prostate cancer: a solution for the growing problem?. Prostate Cancer Prostatic Dis.

[14] Barlow KH, van der Pols JC, Ekberg S (2022). Johnston EA Cancer survivors’ perspectives of dietary information provision after cancer treatment: A scoping review of the Australian context. Health Promot J Austr.

[15] Smith SK, Wiltshire G, Brown FF, Dhillon H, Osborn M, Wexler S, Beresford M, Tooley MA (2022). Turner JE ‘You’re kind of left to your own devices’: a qualitative focus group study of patients with breast, prostate or blood cancer at a hospital in the South West of England, exploring their engagement with exercise and physical activity during cancer treatment and in the months following standard care. BMJ Open.

[16] Ligibel JA, Pierce LJ, Bender CM, Crane TE, Dieli-Conwright C, Hopkins JO, Masters GA, Schenkel C, Garrett-Mayer E (2022). Katta S Attention to diet, exercise, and weight in oncology care: Results of an American Society of Clinical Oncology national patient survey. Cancer.

[17] Baguley BJ, Benna-Doyle S, Drake S, Curtis A, Stewart J, Loeliger J (2024) Access to nutrition services and information after active cancer treatment: a mixed methods study. J Cancer Surviv 18(1):176–185 10.1007/s11764-023-01352-x PMC10866769 36823493

[18] Kennedy MA, Bayes S, Newton RU, Zissiadis Y, Spry NA, Taaffe DR, Hart NH (2022). Galvão DA Implementation barriers to integrating exercise as medicine in oncology: an ecological scoping review. J Cancer Surviv.

[19] Michie S, Van Stralen MM (2011). West R The behaviour change wheel: a new method for characterising and designing behaviour change interventions. Implement Sci.

[20] Sandelowski M (2000). Whatever happened to qualitative description?. Res Nurs Health.

[21] Stanley M, Nayer S, Stanley M (2023). Qualitative descriptive: A very good place to start. Qualitative research methodologies for occupational science and occupational therapy.

[22] Morgan DL (1996) Focus groups as qualitative research. Sage publications, Thousand Oaks

[23] Tong A, Sainsbury P (2007). Craig J Consolidated criteria for reporting qualitative research (COREQ): a 32-item checklist for interviews and focus groups. Int J Qual Health Care.

[24] Mehta LS, Watson KE, Barac A, Beckie TM, Bittner V, Cruz-Flores S, Dent S, Kondapalli L, Ky B (2018). Okwuosa T Cardiovascular disease and breast cancer: where these entities intersect: a scientific statement from the American Heart Association. Circulation.

[25] Wilk M, Waśko-Grabowska A (2020). Szmit S Cardiovascular complications of prostate cancer treatment. Front Pharmacol.

[26] Phillippi J (2018). Lauderdale J A guide to field notes for qualitative research: Context and conversation. Qual Health Res.

[27] Braun V (2006). Clarke V Using thematic analysis in psychology. Qual Res Psychol.

[28] Clarke V, Braun V (2021) Thematic analysis: a practical guide. Sage publications, London

[29] Gildea GC, Spence RR, Jones TL, Turner JC, Macdonald ER, Hayes SC (2023). Sandler CX Barriers, facilitators, perceptions and preferences influencing physical activity participation, and the similarities and differences between cancer types and treatment stages-A systematic rapid review. Prev Med Rep.

[30] Hyatt A, Shelly A, Cox R, Humphries E, Lock G (2022). Varlow M How can we improve information for people affected by cancer? A national survey exploring gaps in current information provision, and challenges with accessing cancer information online. Patient Educ Couns.

[31] Ferri A, Gane EM, Smith MD, Pinkham EP, Gomersall SR (2021). Johnston V Experiences of people with cancer who have participated in a hospital-based exercise program: a qualitative study. Support Care Cancer.

[32] Harvey BI, Youngblood SM (2023). Kleckner AS Barriers and facilitators to adherence to a mediterranean diet intervention during chemotherapy treatment: a qualitative analysis. Nutr Cancer.

[33] Baguley B, Smith-Gillis C, Porter J, Kiss N, Ugalde A (2023) Nutrition services during prostate cancer androgen deprivation therapy. BMJ Support Palliat Care 10.1136/spcare-2023-004304 37402540

[34] Tsai E, Robertson MC, Lyons EJ, Swartz MC (2018). Basen-Engquist K Physical activity and exercise self-regulation in cancer survivors: A qualitative study. Psychooncology.

[35] Spencer JC (2016). Wheeler SB A systematic review of motivational interviewing interventions in cancer patients and survivors. Patient Educ Couns.

[36] Amireault S, Fong AJ (2018). Sabiston CM Promoting healthy eating and physical activity behaviors: a systematic review of multiple health behavior change interventions among cancer survivors. Am J Lifestyle Med.

[37] Baguley BJ, Dalla Via J, Fraser SF, Daly RM (2023). Kiss N Effectiveness of combined nutrition and exercise interventions on body weight, lean mass, and fat mass in adults diagnosed with cancer: a systematic review and meta-analysis. Nutr Rev.

[38] Ligibel JA, Jones LW, Brewster AM, Clinton SK, Korde LA, Oeffinger KC, Bender CM, Tan W, Merrill JK (2019). Katta S Oncologists’ attitudes and practice of addressing diet, physical activity, and weight management with patients with cancer: findings of an ASCO survey of the oncology workforce. J Oncol Pract.

[39] Nadler M, Bainbridge D, Tomasone J, Cheifetz O, Juergens RA (2017). Sussman J Oncology care provider perspectives on exercise promotion in people with cancer: an examination of knowledge, practices, barriers, and facilitators. Support Care Cancer..

[40] Caperchione CM, Sharp P, Phillips JL, Agar M, Liauw W, Harris CA, Marin E, McCullough S (2022). Lilian R Bridging the gap between attitudes and action: A qualitative exploration of clinician and exercise professional's perceptions to increase opportunities for exercise counselling and referral in cancer care. Patient Educ Couns.

[41] Feighan L, MacDonald-Wicks L, Callister R (2023). Surjan Y Practitioner perceptions on the use of exercise and nutritional interventions for patients with breast cancer receiving radiation therapy. J Med Radiat Sci.

