# Supplementary material for: Exercise and diet support in breast and prostate cancer survivors: findings from focus groups
Source: Support Care Cancer. 2024 Jun 18;32(7):440. doi: 10.1007/s00520-024-08652-7 (PMC11189317; doi:10.1007/s00520-024-08652-7)
Supplement: Supplementary file 1 — Supplementary file1 (DOCX 19 KB) [file 520_2024_8652_MOESM1_ESM.docx]

**Question list: Exercise and diet support received during and following cancer treatment**

1. **What are your thoughts about the importance of diet and physical activity for people with cancer or who have had cancer?**

*Possible follow-ups:*

- - *What are those thoughts based on?*
  - *Do you think diet and physical activity is more, less or equally important during treatment as it is once you have completed treatment? Why is this?*
  - *How important do you think it is to receive information and support about diet and physical activity from a healthcare professional?*
  - *Was there anything about your diet or physical activity levels that you wanted to change following your cancer diagnosis or treatment?*
  - What information was provided about why diet/exercise is beneficial specifically in relation to cancer/treatment side-effects?

1. **Since being diagnosed, what advice or support did you receive about your diet?**

*Possible follow-ups:*

- - *How was this delivered to you (by who, and when)?*
  - *How regularly has diet been brought up to you from diagnosis until now?*
  - *How well do you think you understood the advice provided?*
  - *Have you made any changes to your diet based on the advice provided?*
  - *Was there anything you were told or provided that was particularly useful?*
  - *What research, if any, did you do of your own about diet?*

If none, what advice or support would you have liked to receive in relation to diet?

1. **Since being diagnosed, what advice or support did you receive about exercise or physical activity?**

*Possible follow-ups:*

- - *How was this delivered to you (by who, and when)?*
  - *How regularly has this been brought up to you from diagnosis until now?*
  - *How well do you think you understood the advice provided?*
  - *Have you made any changes to your physical activity levels based on the advice provided?*
  - *Was there anything you were told or provided that was particularly useful?*
  - *What research, if any, did you do of your own about exercise?*

If none, what advice or support would you have liked to receive about physical activity?

1. **Are there any aspects of diet and physical activity you would’ve liked to receive advice/support about but did not?**

*Possible follow-ups:*

- - *What type of advice/information would you have liked to receive?*

1. **How you would have liked diet and/or physical activity advice or support to be delivered?**

*Possible follow-ups:*

- - *Can you think of any ways to better convey the importance of diet and physical activity information early on after being diagnosed?*
  - *Who would you most like to receive this advice/support from?*
  - *What do you think the best format for this information would be?*
